# Use of Transgenic Animals in Biotechnology: Prospects and Problems

**Published:** 2013

**Authors:** O. G. Maksimenko, A.V. Deykin, Yu. M. Khodarovich, P. G. Georgiev

**Affiliations:** Institute of Gene Biology of the Russian Academy of Sciences, Vavilov St., 34/5, Moscow, Russia, 119334; Shemyakin-Ovchinnikov Institute of Bioorganic Chemistry of the Russian Academy of Sciences, Miklucho-Maklai St., 16/10, Moscow, Russia, 117997

**Keywords:** bioreactor, milk protein production, production of monoclonal antibodies, recombinant proteins, therapeutic drugs, transgenic animals

## Abstract

During the past two decades, there have been numerous attempts at using animals in order to produce recombinant human proteins and monoclonal antibodies. However, it is only recently that the first two therapeutic agents isolated from the milk of transgenic animals, C1 inhibitor (Ruconest) and antithrombin (ATryn), appeared on the market. This inspires hope that a considerable number of new recombinant proteins created using such technology could become available for practical use in the near future. In this review, the methods applied to produce transgenic animals are described and the advantages and drawbacks related to their use for producing recombinant human proteins and monoclonal antibodies are discussed.

## INTRODUCTION

After the successful expression of the first recombinant proteins (RPs) in bacteria and yeast, it became clear that a large number of human RPs could not be efficiently produced using such systems. Thus, human proteins do not undergo post-translational modifications in bacterial cells, and the nature of the modifications in yeast cells is different from those that take place in human cells. Additionally, these expression systems cannot ensure the proper folding of a number of complex human RPs [1, 2]. Therefore, the research community faced the challenge of developing alternative expression systems capable of ensuring correct post-translational modifications in RPs. A simultaneous development of two technological models (based on transgenic animals and mammalian cell cultures) was started as a result.

**Fig. 1:**
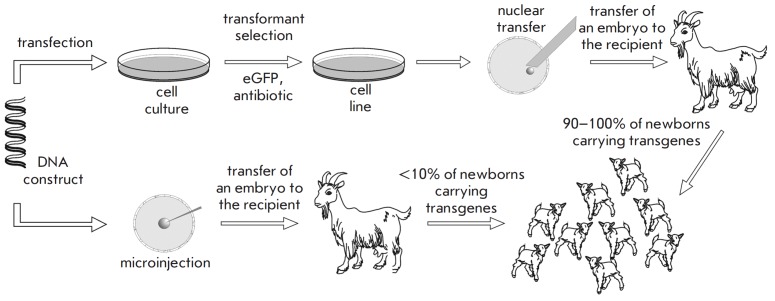
Scheme for producing transgenic animals using the methods of nuclear transfer (upper panel) and intranuclear microinjection of DNA (lower panel)

The first successful production of transgenic mammals by the microinjection of genetically engineered constructs into the pronucleus of a mouse zygote was carried out over 20 years ago [3]. A large number of transgenic animals (TAs) have been produced since for scientific purposes, to improve livestock and to produce RPs [4–9]. Until the end of the past century, TAs had been considered to be the most promising models for producing human RPs and monoclonal antibodies (mAb). Yet, it was mammalian cell cultures (Chinese hamster ovary cells (CHO) in particular) that played the dominant role in the production of RPs. Thus, 312 therapeutic products obtained using living organisms had been introduced to the U.S. market by 2012 [10]. A total of 193 products were obtained using mammalian cell cultures, and 42 of them were produced using CHO cell cultures. This has been largely attributed to the fact that it was not until 2006 that the European Medicines Evaluation Agency (EMEA) approved antithrombin, the first recombinant protein derived from the milk of transgenic goats [11]. This protein was subsequently approved for commercialization by the United States’ Food and Drug Administration (FDA) as a drug that prevents blood clotting in patients with hereditary antithrombin deficiency. In 2011, the EMEA approved the use of the recombinant C1-esterase inhibitor produced in rabbits for the treatment of hereditary angioedema. The arrival on the market of the first therapeutic products produced using TAs and their approval for medical use suggest that RPs could carve out a significant niche in biotechnology in the near future. Some biotechnology companies (PPL Therapeutics (England), GTC Biotherapeutics (USA) (acquired by LFB Biotechnologies, France, in 2010), Hematech (USA), Genzyme (USA), ZymoGenetics (USA), Nexia Biotechnologies (Canada), Pharming (Netherlands), BioProtein Technologies (France), Avigenics (USA), Viragen (USA), and TranXenoGen (USA)) are actively working on developing this technology. This review discusses the general concepts behind generating TAs for the production of human RPs and mAb.

## THE MAIN METHODS USED TO PRODUCE TRANSGENIC ANIMALS

The contemporary methods that allow one to obtain RP-producing livestock that contain the required transgene in all the cells of their organism and pass it on to their offspring include intra-pronuclear zygotic DNA microinjection (MI) and somatic cell nuclear transfer (NT ). Today, DNA microinjection into the male pronucleus of a zygote is the most commonly used method [12] (*Fig. 1*). As it enters the nucleus, linear DNA is capable of integrating into the genome of cell lines or living organisms [13]. DNA is usually integrated into transcriptionally inactive gene-poor regions and into heterochromatin. From one to several or even hundreds of copies of the injected construct can integrate into one genomic site. This technology was initially tested on mice, and it remains a reliable method for the production of TAs. This method was used to produce the first agricultural TAs. However, MI is now used mainly to produce transgenic mice, rabbits and pigs. This is attributed to the insufficient efficiency of the method due to the low frequency of incorporation of recombinant DNA into the genome and the availability of zygotes at the two pronuclear stages. The result depends on carrying out a large number of surgical procedures, which entails the need to keep a substantial number (200–300 heads) of experimental livestock and perform skilled animal handling. Furthermore, the only way to determine the expression level of the integrated transgene is to examine the original TAs and their offspring. The reproductive cycle in large animals (including the time before they reach physiological maturity and the need to obtain females producing RPs in milk from the original transgenic males) is approximately 0.9/2.3 years for goat females/males, 1.0/2.3 years for pigs, and 2.3/4.5 years for cows. These limitations increase the cost of obtaining the original TAs and the time required to organize the work.

In 1997, a sheep clone was produced by nuclear transfer (NT ) of a somatic mammary gland cell into an oocyte [14]. This achievement opened the possibility of developing cheaper and easier procedures for producing agricultural TAs (*Fig. 1*), since most of the manipulations in this case are moved from a farm to a laboratory, where the transfection of somatic cells is carried out and clones characterized by the integration of the transgene into the genome are selected. The nucleus of the somatic cell is then injected into the enucleated oocyte, which is transplanted into female recipients. Fibroblast cells are typically used for NT. The majority of recently generated large farm animals have been obtained by NT [12]. However, the transfected cells in this case are selected using antibiotic resistance marker genes, which complicates the approval of the produced recombinant proteins by the FDA and EMEA [15]. Fluorescent proteins, such as the enhanced green fluorescent protein (eGFP), are often used as an additional selective agent in order to increase efficiency in such selection [16]. Systems based on site-specific recombinases are additionally used to remove selection markers from the genome of the selected cell lines [17].

The adverse effects of the NT technique include a low *in utero *embryo survival rate and poor health of the newborn animals [12]. This is attributed, among other things, to incomplete reprogramming of the somatic nucleus, resulting in impaired expression of several of the genes required for the proper progression of embryogenesis. Moreover, the process of obtaining suitable oocytes and their activation requires considerable expenditures of time and financial resources. As a result, one of the world leaders in the use of NT for the production of agricultural TAs, the AgroResearch company (New Zealand), has rejected the method. The company is now developing alternative methods for producing agricultural TAs.

The site-specific transgenesis technology using embryonic stem (ES) cells could be an alternative to the MI and NT methods [18]. This method involves the insertion of a transgene into the genome of ES cells, followed by the selection of clones with a proper integration of the required number of copies, before transgenic ES cells are introduced into the cavity of a blastocyst, which is transplanted into a recipient female. After these cells are transplanted into the ovaries of adult mice, up to 30% of newborn mice can carry the transgene. All animal handling can be performed using nonsurgical methods, which are widely used in animal husbandry. The production of transgenes requires a relatively small number of blastocysts and, hence, a small experimental herd. However, this method has only been perfected for mice and rats; ES cell lines for farm animals have yet to be obtained. A similar approach involves the transformation of stem cells, the precursors of sperm cells, and their subsequent transplantation into seminiferous tubules of infertile males [19].

The other methods for obtaining TAs are relatively rarely used. Thus, TAs can be effectively produced using retroviruses containing the required transgene [12]. In order to achieve this objective, the zygotes lacking protective coating are cultured in a medium supplemented with lentiviral particles, followed by transplantation into female recipients. The integration of one to several copies of the transgene occurs depending on the lentiviral titer; almost 100% of the offspring can be transgenic in this case [12]. The advantages of this method include efficient production of any species of TAs and the opportunity to produce TAs carrying only one transgene copy, which is sometimes necessary for scientific purposes. The main drawbacks of the method include the inability to use the introns present in the gene construct and the limitation of the transgene length (approximately 8000 bp), which is determined by the size of the viral particle. As a result, it is very difficult to achieve a high level of transgene expression using this method.

A promising method for obtaining TAs is the use of vectors based on mobile genetic elements, which are integrated into the genome by transposase [12]. The gene encoding transposase and the transgene flanked by terminal repeats of transposon are coinjected into the zygote. The reaction catalyzed by transposase results in the integration of a single copy of the transgene into one or several sites of the animal’s genome. This approach has been used to produce large farm animals (e.g. pigs [20]). The efficiency of the integration of the transgene in this case depends on the type of transposon, transgene length, concentration and site of DNA injection, and can be as high as 50% [20]. However, no data regarding the levels of expression of the target gene in the TAs produced using this method have been obtained thus far.

The group of methods based on infecting the organs or tissues of an organism with a replication-defective adenovirus containing the gene of the target protein should be specifically mentioned. This approach results in a short-term nonhereditary production of RPs in the organ or tissue under consideration.

Today, it remains difficult to compare the efficiency of new and traditional methods for producing TAs.

## THE EXPRESSION VECTORS USED TO OBTAIN TRANSGENIC ANIMALS

**Fig. 2:**
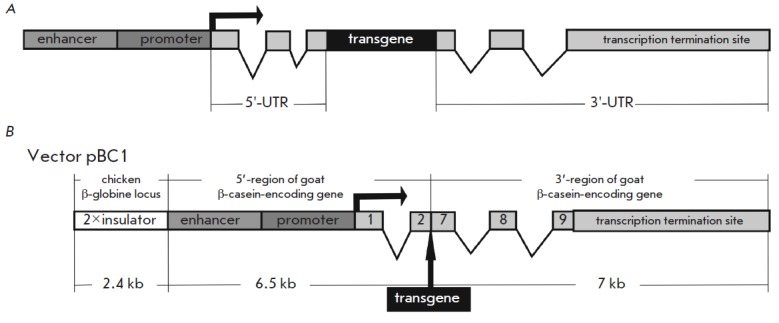
Vectors used for the production of recombinant proteins. (A) Vector structure used in the production of recombinant proteins in transgenic animals and cell lines. (B) Structure of the pBC1 vector used for the production of recombinant proteins in the milk of transgenic animals

The expression vectors used to produce RPs in milk contain regulatory regions of genes whose protein products comprise the major fraction of milk. The most popular examples of the latter include the regulatory regions of the lactoglobulin sheep gene, the acidic protein gene of rodents (mouse, rat) and rabbit, the α-lactalbumin and α-S1-casein genes of cow, and the goat β-casein gene [5]. An expression vector typically includes a long 5’-region (1–7 kb) which consists of a promoter, tissue-specific enhancers that increase the expression in mammary glands, the first non-coding exons and introns located between them (*Fig. 2A*). The first introns of the genes are likely to contain the regulatory elements which can enhance gene transcription. The expression vector also includes the 3’-untranslated region (UTR ) of a gene, whose size can vary from 0.5 to 10 kb and even more. The 3’-UTR typically includes the last non-coding exons and introns, a polyadenylation site, and the adjacent sequences, which have the potential to enhance transcription termination. The 5’- and 3’-UTR s in a vector may belong to either one or different genes.

Among the promoters used for expression in mammary glands, the β-casein gene promoter is one of the most efficient and is used for the production of target proteins in the mammary glands of mice, goats and cows (*Table 1*). The most popular commercial vector for the production of RPs in the milk of TAs – pBC1 (Invitrogen) – was produced using the aforementioned promoter (*Fig. 2B*). This vector allowed one to obtain most of the transgenic goat lines characterized by a high level of target protein production. The regulatory region of the β-casein gene with a length of 6.2 kb and consisting of a promoter and a hormone-dependent enhancer that stimulates the promoter only in the mammary gland cells is used in this vector [31]. The structure of the vector also includes a 7.8-kb-long 3’-region of the β-casein gene, which ensures efficient transcription termination. The latter is required for the formation of a stable mRN A encoding the target protein and for the prevention of the transcription of the adjacent genomic regions capable of causing the formation of repressed chromatin by RN A interference. In order to accumulate the target protein in milk, the coding region of the gene must contain the signal peptide sequence required for secretion. This sequence can be obtained from any gene encoding the secreted protein.

Depending on the aim, either a complete gene with introns or its cDNA or a mini-gene containing only some of the introns is inserted into the vector. The use of a gene with an unmodified exon–intron structure allows one to obtain much higher levels of target protein production in TAs in comparison with the use of cDNA [32, 33]. For instance, the human lactoferrin gene was expressed using the same vector, pBC1, in several independent studies (*Fig. 2B*). The concentration of recombinant lactoferrin did not exceed 4 mg/ml of mouse milk and 0.7 mg/ml of transgenic goat milk if cDNA was used to produce the TAs (*Table 1*). Transgenic mice were produced using the native lactoferrin gene with introns (50 kb long). The concentration of recombinant lactoferrin in their milk was as high as 160 mg/ml (*Table 1*). Transgenic goats carrying one copy of the construct but expressing up to 10 mg/ml of the recombinant human lactoferrin in their milk have also been produced [22]. The difference in the expression of recombinant lactoferrin using the αS1-casein promoter of cows was similar (*Table 1*). This example demonstrates that the presence of introns in the coding region of the transgene results in a two-fold increase in the amount of the target protein in milk.

**Table 1:** Comparison of production of human milk recombinant proteins (RPs) in transgenic animals (TAs) using various variants of the gene construct

RP/construct	Regulatory elements	TA/method of production	Maximum level of RP production, mg/ml	Reference
Lactoferrin				
Native gene	Bacmid	Cow/NT	3.4	[16]
Native gene	WAP-gene (21 kb) (mouse)	Mouse/MI	30	[21]
–“–	β-casein promoter (goat) + insulator	Mouse/MI	160	[22]
–“–	β-casein promoter (goat) + insulator	Goat/MI	10.8	[22]
–“–	αS1-casein promoter (cow)	Cow/MI	3	[23]
cDNA	β-casein promoter (goat) + insulator	Goat/MI	0.7	[24]
cDNA	αS1-casein promoter (cow)	Mouse/MI	0.036	[25]
–“–	β-casein promoter (goat) + insulator	Mouse/MI	4	[26]
Lysozyme				
cDNA	β-casein promoter (goat) + insulator	Cow/NT	0.026	[17]
–“–	β-casein promoter (goat) + insulator	Pig/NT	0.00032	[27]
–“–	α-S1- casein (cow)	Goat/MI	0.27	[28]
–“–	α-S1- casein (cow)	Mouse/MI	0.00071	[29]
α-Lactalbumin				
Native gene	Bacmid	Cow/NT	1.55	[30]

The site at which the construct is integrated into the genome plays a crucial role in ensuring efficient transgene expression. Injected DNA is typically incorporated into the gene-poor regions, which are characterized by frequent DNA breaks [13]. The chromatin in these regions typically exerts a negative influence on the expression of the transgene integrated nearby. In addition, several copies of the construct are typically integrated onto the same genomic site, which can, in turn, lead to repression of transcription due to the formation of heterochromatin in repetitive sequences.

A number of regulatory elements are used to protect the transgene expression from repression and to maintain the direct relationship between the number of copies and the level of transgene expression in mammalian cell cultures: A/T-rich regions of DNA, which bind to the nuclear matrix fraction (known as MAR/SARelements) [34, 35]; regulatory elements (UC OE) that activate the promoters of the “household” genes [36]; STAR-elements that can block the spread of heterochromatin [37]; and insulators [38, 39].

Among the mentioned regulatory elements, only insulators are used in vector constructs to produce TAs. Insulators are the regulatory elements that block the interaction between an enhancer and a promoter, if located between them [40, 41]. Moreover, some insulators can act as a boundary between the transcriptionally active chromatin and heterochromatin. The insulator from a cluster of chicken β-globin genes (HS4 insulator) is one of the most intensely studied vertebrate insulators. It is 1200 bp long and is located at the 5’-end of the β-globin locus [42]. A 250-bp-long core region characterized by full insulator activity has been found in it. This segment contains the binding site for the CTC F protein, which is the only characterized vertebrate insulator protein [43]. The CTC F protein is responsible for the ability of the HS4 insulator to block enhancers. It also assists the USF1 and USF2 proteins (which form the boundary between active chromatin and heterochromatin) to bind to the insulator [44]. The HS4 insulator sequence also binds to the BGP1/Vezf1 protein [45], which protects the GC-rich sequences of an insulator against methylation, which leads to a disruption of the binding of insulator proteins to DNA and, as a result, to inactivation of the insulator. According to the existing model, BGP1/Vezf1 also terminates the weak transcription initiated in the heterochromatic region, which can play an important role in protecting the β-globin locus against the propagation of inactive chromatin [46]. The pBC1 vector constructed by Invitrogen (USA) for TAs production contains two 1.2-kb-long copies of the HS4 insulator at the 5’-end of the vector (*Fig. 2B*). A thorough analysis of the effect of the HS4 insulator on the transcriptional activity of several promoters, including the goat β-casein promoter and rabbit WAP-promoter, demonstrated that the insulator significantly increases the level of transgene expression and the number of transgenic lines that are characterized by a significant production of the target protein [22, 47–50]. Meanwhile, the HS4 insulator neither affects the variability of the transgene expression and its ectopic expression in other tissues of the organism nor ensures a direct correlation between the number of transgene copies and its level of expression. Thus, the HS4 insulator acts as a universal transcription regulator that can be used to increase the activity of weak promoters. However, it does not allow to achieve efficient transgene expression exclusively in a mammary gland, which is important for the production of many target proteins that can adversely affect the health of TAs.

Increasing the size of the regulatory sequences in the transgene construction can be an alternative to insulators and other regulatory elements. Multiple loci expressing milk protein genes possess extended 5’- and 3’-regions, which can contain both tissue-specific enhancers and insulators capable of providing protection against the influence of adjacent genes. For instance, a 50-kb-long construct was synthesized in which the coding region (3 kb) of the mouse WAP gene consisting of 24 kb was replaced with a structural part of the human lactoferrin gene (29 kb) [21]. As a result, transgenic mice have been obtained whose mammary glands are characterized by high tissue-specific transgene expression, and the production of recombinant human lactoferrin in their milk was as high as 30 mg/ml (*Table 1*).

Another method for obtaining TAs that efficiently produce target proteins is the integration of large DNA segments (up to 250 kb) into the genome. Vectors based on bacterial artificial chromosomes (bacmids), which enable cloning of sequences up to 400 kb long, can be used to prepare these extended gene constructs [51, 52]. The regulatory regions of tissue-specific genes can occupy large genomic regions and be a part of the neighboring genes. For example, several enhancers that stimulate the pig WAP gene are found 140 kb away from the gene they regulate and are separated from it by the other genes [53]. When large DNA fragments are used, it is highly likely that all of the regulatory elements of this gene are included in the transgene. It is assumed that the use of this approach results in specific transgene expression exclusively in the mammary gland and that the influence of the surrounding chromatin on transgene expression is minimized. This approach allows one to obtain TAs whose level of transgene expression closely corresponds to the expression of the endogenous counterpart. For instance, transgenic cows expressing the genes of human lactoferrin and human α-lactalbumin have been produced (*Table 1*). In general, this method allows one to achieve stable transgene expression at a level similar to that of native genes. Thus, the levels of production of lactoferrin and alpha-lactalbumin in transgenic cows were 3.4 and 1.55 mg/ml, respectively (*Table 1*). The problem is associated with the other genes, which are a part of the bacmid structure and whose expression can adversely affect the health of the TAs. It should also be mentioned that the use of a bacmid does not completely suppress the effect of the genomic surrounding: the expression is partially dependent on the genome integration site [54, 55]. In this case, there is no direct observable relationship between the number of bacmid copies and the expression level. This can be attributed to the fact that the initiated RN A interference adversely affects the gene expression in a bacmid.

## PRODUCTION OF HUMAN RECOMBINANT PROTEINS USING TRANSGENIC ANIMALS

Since the early 1990s, attempts have been made to produce TAs that synthesize a variety of human proteins. Today, these proteins are produced in other expression systems (bacteria, yeast, mammalian cells). Most of the recombinant human proteins that are produced in mammalian cell cultures are plasma proteins [56]. The use of recombinant plasma proteins grows every year as the scope of their application expands and the use of human tissues for isolating native proteins is constrained by the existing risk of viral contamination, the small number of donors, and ethical considerations. The coagulation factors VII, VIII and IX are used for lifelong treatment of hereditary diseases. An immune response to therapeutic agents develops in most patients over time, despite the highly efficient purification of the proteins produced in bacterial or yeast systems. This fact creates the need for replacing the drug with an analog produced in a different manner. Therefore, the production of recombinant coagulation factors in the milk of TAs is of significant medical importance [57]. *Table 2* shows some examples of TAs whose milk contains human blood clotting factors. Treatment of blood diseases in most cases requires a comparatively small amount (calculated in grams) of RPs. Consequently, a rabbit is the optimal TA for producing RPs: each transgenic rabbit female can produce approximately 5 liters of milk per lactation or 20 g of RPs per year. The results obtained demonstrate that the expression of coagulation factors does not affect animal health and lactation [74, 75].

**Table 2:** Examples of the expression of human recombinant proteins (RPs) in the milk of transgenic animals (TAs)

RP (construct)	Regulatory elements	TA/method of production	Maximum level of RP production in milk, mg/ml	Reference
Albumin (native gene)	β-casein promoter (goat) + insulator	Cow/NT	40	[15]
α-fetoprotein (native gene)	β-casein promoter (goat) + insulator	Goat/NT	1.1	[58]
Butyrylcholinesterase (cDNA)	–“–	Goat/NT	5	[59]
Granulocyte colony-stimulating factor (native gene)	–“–	Goat/NT	0.05	[60]
Growth hormone (native gene)	β-casein promoter (goat)	Goat/NT	0.07	[61]
Antithrombin (cDNA)	β-casein promoter (goat)	Goat/MI	2	[62]
Coagulation Factor IX (mini-gene)	–“–	Mouse/MI	0.026	[63]
Tissue plasminogen activator (cDNA)	–“–	Goat/MI	3	[64]
Coagulation Factor IX (cDNA)	β-casein promoter (cow)	Goat/MI	9.5 × 10^-5^	[65]
Growth hormone (native gene)	β-casein promoter (cow)	Cow/NT	5	[66]
Granulocyte colony-stimulating factor (native gene)	α-S1- casein promoter (goat)	Mouse/MI	0.04	[67]
Erythropoietin(cDNA)	β-lactoglobulin promoter (cow)	Mouse/rabbit/MI	0.3 (mouse)	[68]
			0.5 (rabbit)	
Lysostaphin (native gene)	β-lactoglobulin promoter (sheep)	Cow/NT	0.014	[69]
Lysostaphin (cDNA)	β-lactoglobulin promoter (sheep)	Mouse/MI	1.3	[70]
C1-esterase inhibitor (native gene)	WAP-promoter (mouse)	Rabbit/MI	1.8	[71]
Coagulation Factor IX (cDNA)	WAP-promoter (mouse)	Pig/MI	4	[72]
Coagulation Factor VIII (cDNA)	WAP-promoter (mouse)	Rabbit/MI	0.1	[73, 74]

Unlike the coagulation factors VII, VIII and IX, the demand for recombinant albumin is calculated in tons, since albumin is used not only in medicine, but also in biotechnology to stabilize other proteins. Albumin is the major blood protein, which is usually isolated from plasma. The production of recombinant albumin is more expensive than its isolation from blood plasma, since a very high degree of purification is required for its medical application. Nowadays, recombinant albumin is produced mostly in yeasts *Saccharomyces cerevisiae* (Recombumin^TM^) and *Pichia pastoris *(Albrec^TM^). The huge demand for recombinant albumin has determined the choice of transgenic cows for its production. Thus, GTC Biotherapeutics (USA) has recently created transgenic cows whose average level of recombinant human albumin (rhAB) production in milk is 1–5 mg/ml [15] or up to 30 kg per producing cow per year. The same work described a line of transgenic cows whose RHA concentration in milk was as high as 48 mg/ml, which corresponds to the integration of 250 copies of the construct. Transgenic cows of this line are characterized by a shorter period of lactation and a decrease in the milk yield. Thus, it can be assumed that the production of rhAB in the milk of transgenic cows must be below 48 mg/ml.

Certain proteins, such as hormones and cytokines, have a negative effect on the lactation and health of TAs. This makes maintenance of the transgenic herd problematic. The most notable project undertaken by the PharmAthene Inc. company (USA) on the instructions of the Ministry of Defense is connected with the production of butyrylcholinesterase (*Table 2*), a highly active enzyme that efficiently protects against organophosphate poisons. As a result, a herd of goats has been produced whose level of production of recombinant human butyrylcholinesterase (rhBChE) in milk is 1–5 mg/ml [59]. The main problem the company encountered was the effect of rhBChE on lactation. The latter significantly reduced the productivity of transgenic goats [76]. As a result, a question regarding the economic feasibility of using transgenic goats to obtain rhBChE has arisen.

There are several approaches that allow one to produce RPs that adversely affect lactation and the health of the TAs; however, they only resolve the problem partially. First of all, a promoter that stably functions only in the mammary gland at a relatively low level can be selected. For instance, a recombinant human granulocyte colony stimulating factor (rhG-CSF) was produced using the β-casein gene promoter without an enhancer in transgenic goats (*Table 2*) [60]. However, the rhG-CSF concentration in the milk of the goats did not exceed 0.05 mg/ml. Transgenic mice with milk containing 0.02–0.04 mg/ml of rhG-CSF have also been produced. An expression vector containing the 5’-regulatory region of the *CSN1S1 *gene of a goat (3387 bp), including the first intron and the 3’-region of the *CSN1S1 *gene of a cow (1518 bp) with non-coding exons 18 and 19, was also used [67]. As a result, it was demonstrated that transgenic mice carrying this vector express rhG-CSF exclusively in milk, but not in other tissues. However, the low level of RPs in the milk reduces the economic attractiveness of this approach.

An alternative way to produce RPs, which adversely affects the health of the producing TAs, is infection of a mammary gland with replication-defective vectors based on adenoviruses. Thus, an adenoviral vector designed to express recombinant human erythropoietin was produced at the Laboratory of Transgenesis and Animal Cloning (Havana, Cuba). The erythropoietin concentration in the milk of the goats infected with this adenovirus reached 2 mg/ml, but it exhibited a low biological activity, which was presumably due to insufficient glycosylation of the protein produced using this approach [77]. The production of the recombinant human growth hormone in mice (2 mg/ml) and goats (0.3 mg/ml) using adenoviral vectors has been described [78]. A similar approach was used in the case of recombinant human lactoferrin, whose concentration in the milk of goats was as high as 2 mg/ml [79]. Despite the simplicity of using an animal adenoviral vector to create TAs expressing the target protein in milk, this method does not allow one to obtain a stable expression of the recombinant protein at a level sufficient for its commercial production. A high expression level (1.5–2 mg/ml) was observed exclusively during the first 25 days of lactation, which can be explained by either natural death of the transfected cells or the immune response to the infection.

Finally, the production of the inactive forms of proteins is considered to be a promising approach. For instance, an expression vector containing erythropoietin cDNA integrated into the fifth exon of the lactoglobulin gene of a cow [68] in such a manner that there was a region cleavable by IgA-protease between the coding regions of two genes was constructed in order to produce recombinant human erythropoietin. As a result, transgenic mice and rabbits were produced in whose milk the concentration of chimeric protein reached 0.3 and 0.5 mg/ml, respectively. Following the cleavage of the chimeric protein by IgA-protease, the activity of erythropoietin was restored and lactation and the health of the TAs were not affected. It is also possible to use the co-expression of RPs and an inhibitor that blocks its activity. Thus, the recombinant human prourokinase expressed in milk almost immediately transforms into its active form, urokinase, which makes this bioreactor unpromising with respect to the production of a therapeutic form of the protein (prourokinase). Prourokinase was co-expressed with the bacterial serine protease inhibitor in the milk of transgenic mice in order to resolve this problem [80]. This allowed to purify the milk of transgenic mice from the processed prourokinase (urokinase) and to dramatically increase the yield of the therapeutic form of the protein.

It should be mentioned that sialylation of RPs in the milk of transgenic rabbits and pigs is most similar to sialylation in human cells, which is essential for reducing the immunogenicity of the drugs used in long-term therapy [81, 82]. Incorrect post-translational modifications that reduce the activity of the recombinant protein can occur in the milk of transgenic goats and cows. The easiest way to remove the incorrect modification is mutation in the protein site where the undesired modification occurs. For example, alpha-fetoprotein, a single chain glycosylated plasma protein with a molecular weight of 68 kDa, is used to treat autoimmune diseases. The demand for a properly folded recombinant human alpha-fetoprotein (rh-AFP) is extremely high (kilograms of protein are needed); hence, the Merrimack Pharma company (USA), together with GTC Biotherapeutics (USA), have launched a project for the production of transgenic goats that produce rh-AFP in their milk. The human alpha-fetoprotein isolated from the milk of transgenic goats was glycosylated at the asparagine residue located at position 233, which greatly reduced its activity. Therefore, the glutamine residue replaced the asparagine residue in rh-AFP, which caused inactivation of the glycosylation site [58, 83]. It was demonstrated that the biological activity and pharmacokinetics of the mutant variant of alpha-fetoprotein are similar to those of the native protein.

The mAb market is the fastest growing segment of the pharmaceutical industry. Therapeutic mAbs, most of which are used to treat cancer and autoimmune diseases, generated profit of over $26 billion for American biotechnological companies in 2007 [84].

The mAbs currently used in medicine are produced exclusively in mammalian cell cultures, since proper post-translational modifications are required to ensure therapeutic efficiency. The most important modifications include the attachment of oligosaccharides and sialic acid, which considerably increase the mAbs bloodstream circulation time and reduce their immunogenicity. However, the RPs produced in cell cultures have a relatively high cost. Hence, an attempt to use TAs to produce antibodies was made at the end of the 1990s [85, 86]. Since mAbs are composed of two polypeptide chains, two constructs containing the genes encoding heavy and light subunits were used for their expression in TAs. When producing TAs, several constructs encoding the heavy and light chains of the antibody are typically incorporated into the same genomic site. During the initial experiments, mAbs were expressed using various gene promoters of milk proteins, e.g., sheep β-lactoglobulin [87] and mouse WAP [88, 89]. Transgenic mice whose milk contains mAbs at relatively high concentrations of 0.4–5 mg/ml have been produced as a result (*Table 3*). mAbs for pharmaceutical production were subsequently obtained from transgenic goats; mAbs for testing expression vectors, evaluating the quality of mAbs, and refining the methods used for their isolation were obtained from transgenic mice. The pBC1 vector described above has been widely used for the expression of mAbs (*Fig. 2B*). The highest expression levels of mAbs in transgenic mice (as high as 32 mg/ml of milk) were obtained using this vector (*Table 3*). However, published data on the expression level of mAbs in the milk of transgenic goats are virtually absent. Transgenic goats, one of which showed an expression level approaching 14 mg/ml, have been mentioned in only one review [86].

**Table 3:** Examples of mAb production in the milk of transgenic animals (TAs)

Antibody binding antigen	Regulatory elements	TA/constructs	Maximum level of antibody production in milk, mg/ml	Reference
CD6-receptor	WAP-promoter (rabbit)	Mouse/two native genes	0.4	[90]
Envelope glycoprotein S (gastroenteritis coronavirus)	WAP-promoter (mouse)	Mouse/two native genes	5	[89]
Envelope glycoprotein S (gastroenteritis virus)	β-lactoglobulin promoter (sheep)	Mouse/two cDNA	6	[91]
BR96 anti-Lewis Y	β-casein promoter (goat)	Mouse/two native genes	14	[86]
BR96 anti-Lewis Y	β-casein promoter (goat)	Mouse/two native genes	4	[86]
CD20-receptor	β-casein promoter (goat) + insulator	–“–	22.3	[92]
Surface antigen (Hepatitis A virus)	β-casein promoter (goat) + insulator	–“–	32	[93]
Surface antigen (Hepatitis B virus)	–“–	–“–	17.8	[94]

According to the data provided by the GTC Biotherapeutics company (USA), mAbs isolated from the milk of transgenic goats are typically stable and highly efficient; even high levels of mAb expression do not affect the health and lactation of transgenic goats. The company has developed relatively simple methods for obtaining highly purified mAbs that are suitable for medical applications [95, 96]. The conducted investigations led to regarding transgenic goats as the optimal model for the production of mAbs [97]. The attractiveness of transgenic goats is attributed to the fact that they rarely get infected with BSE, in comparison with sheep and cows. Transgenic goats from New Zealand or Australia are currently being used for the production of RPs in milk, since it is officially believed that there is no cow disease in these countries.

The demand for mAbs recently hit the several-hundred- kilograms-per-year mark. For example, world demand for anti-receptor CD20 mAbs exceeds 600 kg per year. It is estimated that a herd consisting of 210 transgenic goats whose milk contains mAbs at a concentration of 8 g/l can fully meet the world demand in anti-CD20 mAbs at an approximate cost of $100/g [85]. Meanwhile, 51,000 l of cell culture with the capacity of 1g/l and an approximate cost of $300/g are required to obtain an equal amount of mAbs.

Despite the relatively low cost of mAb production in transgenic goats, there are some drawbacks associated with their use in comparison with the use of mammalian cell cultures. Firstly, the antibodies must be properly glycosylated and sialylated, which is important for their stability, immunogenicity, and biological activity. Sialylation and glycosylation occur in the mammary glands of transgenic goats, but it may be incomplete. In addition, an increase in the level of mAb expression is associated with a decrease in glycosylation efficiency. Therefore, 2–4 mg/ml is considered to be an optimal level of mAbs in milk. The second problem is associated with the fact that sialic acid is present in transgenic goats in the form of N-acetylneuraminic acid (NANA) [98], while human antibodies contain Nglycolylneuraminic acid (NGNA). There is a possibility that antibodies containing the “wrong” sialic acid could be immunogenic to patients in some cases. Recombinant proteins in mammalian cell cultures also undergo heterogeneous glycosylation and sialylation. These processes are usually not completely identical to their native counterparts. In order to overcome this hurdle, additional genes encoding transporters and enzymes, which increase the level of glycosylation and sialylation, and/ or genes whose RN A-product induces inactivation of the genes encoding the proteins that adversely affect glycosylation, are introduced into the cell lines producing the recombinant proteins [99, 100]. An opportunity to inactivate the genes involved in the glycosylation of RPs in the cell lines, which is different from glycosylation in human cells, recently became available due to the development of the new technologies of site-directed mutagenesis. Similar approaches cannot be used for producing animals, since the changes in the genome can adversely affect the viability of the TAs. The only potential option is to create TAs using a vector under strict control of expression exclusively in the mammary gland. This vector must express additional genes which increase/modify glycosylation and genes that encode groups of RN A capable of inactivating the genes whose protein products are responsible for the abnormal glycosylation of RPs. Finally, the presence of approximately 0.3–0.5 mg/ml of endogenous immunoglobulins in goat milk poses an additional problem during mAb purification. Therefore, an efficient chromatographic separation of goat and human immunoglobulins is required in order to obtain highly purified mAbs [86]. Meanwhile, the introduction of synthetic media for the cultivation of cell cultures significantly simplifies the stage of recombinant protein purification, which somewhat reduces the cost of obtaining highly purified mAbs.

It has recently been demonstrated that the absence of fucose in the glycol chain of an antibody results in an induction of cytotoxicity at an antibody concentration ten times lower than in the case when conventional antibodies are used [101]. A model based on transgenic rabbits could be cost-effective for producing these defucosylated antibodies. Interestingly, no fucose residues have been identified in the recombinant human C1- inhibitor isolated from the milk of transgenic rabbits, suggesting the absence of active fucosylation in rabbit mammary glands. Thus, transgenic rabbits can become an attractive model for the production of this new class of highly active antibodies.

A large-scale project to obtain mAbs in TAs was initiated in the late 1990s by Genzyme Transgenic company (currently known as GTC Biotherapeutics), which signed contracts with a large number of companies developing mAbs as therapeutic agents. During the initial stage, mAbs were obtained in transgenic mice in order to assess their overall activity. If the levels of expression and biological activity of mAbs in transgenic mice were comparable to the expected values, goats-producers were created during the second stage. GTC Biotherapeutics is currently developing a technology for the production of several widely used mAbs (Rituximab^®^, Herceptin^®^, Humira^®^, and Erbitux^®^) in transgenic goats. Work aimed at producing RPs in transgenic goats is being actively conducted in China and New Zealand.

## POTENTIAL ROLE OF TRANSGENIC ANIMALS IN AGRICULTURE

At the moment, the FDA is nearing approval for salmon which expresses the growth hormone for commercial use (AquAdvantage) (according to the findings, it is safe for humans and the environment) [11, 102]. The economic impact in the case of transgenic salmon is associated with an almost twofold increase in growth, which significantly reduces the cost of cultivation. Therefore, it can be assumed that in the near future permission for the commercial application of various TAs will be obtained. These TAs can be used to achieve such important objectives as 1) producing modified milk containing human RPs; 2) altering the composition of milk to increase efficiency in dairy products production; 3) improving the characteristics of farm animals (fast growth, recycling); and 4) improving the resistance of farm animals to bacterial, viral, and prion infections [103].

The issues of artificial infant feeding and nutrition of newborns is gaining in importance. In terms of its composition, breast milk is significantly different from goat and cow milk. Thus, human milk contains much higher concentrations of lactoferrin (2.0–5.8 mg/ml), lysozyme (0.03–3 mg/ml), and lactalbumin (1.8–3.1 mg/ml). These proteins protect the organism against infections, improve the structure of the intestinal epithelium, have a positive effect on the intestinal microflora, and enhance immunity. Meanwhile, the concentration of these proteins is significantly lower in cow milk: 0.03–0.49 mg/ml for lactoferrin, 0.05–0.22 mg/ml for lysozyme, and 1.47 mg/ml for lactalbumin. The mixtures for artificial feeding produced using animal milk do not provide optimal infant nutrition, since they are prepared from hydrolysates and contain no functional proteins.

Transgenic cows expressing recombinant lactoferrin (3.4 mg/ml) [16], lysozyme (0.03 mg/ml) [17] or human lactalbumin (1.5 mg/ml) [33] have been obtained in China to produce modified milk. Milk containing all three human RPs at the optimal concentration is intended for production next. Transgenic goats whose milk contains recombinant human lysozyme at a concentration of 0.27 mg/ml [31] (corresponding to 67% of the lysozyme concentration in breast milk) have been obtained in the USA. It was demonstrated that pasteurized milk with human lysozyme has a positive effect on the health of young goats and pigs [104, 105]. The University of California, Davis (USA), and the Institute of Biomedicine of the Federal University of Ceara (Brazil) received a grant from the Government of Brazil to explore the possibility of using milk containing recombinant human lysozyme to treat diarrhea in children from low-income families. Production of transgenic goats expressing recombinant lactoferrin in milk for the subsequent production of milk simultaneously containing a combination of two human proteins is also scheduled.

The government-owned AgroResearch company in New Zealand produces transgenic cows with the aim of increasing efficiency in cheese production. Caseins, the most valuable proteins, comprise approximately 80% of milk proteins. The casein fraction in cow milk consists of α-S1-, α-S2-, β- and k-caseins, encoded by a single copy of each gene [106]. Caseins aggregate into large micelles. The micellar structure and its stability may vary depending on the ratio of caseins, which affects the physical and chemical properties of the milk. Cheese is made via the aggregation of casein micelles, which retain water and fat by forming a protein network. An increased content of β- and k-caseins leads to a reduction in the micellar size and increases thermal stability, which is necessary for cheese production [107]. In order to increase the amount of β- and k-caseins in milk, transgenic cows with additional copies of the gene were produced [108]. The endogenous β-casein gene of cows with its regulatory sequences was used. Since the k-casein gene is characterized by a relatively low expression level, a chimeric gene containing the regulatory region of the β-casein gene and the coding region of the k-casein was used for the production of transgenic cows. The transgenic cows were eventually produced; their milk was characterized by a 20% increase in the level of β-casein expression and a twofold increase in k-casein synthesis. This result clearly demonstrates the fact that the milk content can be altered by transgenesis, which can increase efficiency in the multi-billiondollar cheese production industry.

One of the problems in swine breeding is the high mortality of piglets attributed to the insufficient content of α-lactalbumin in milk. In order to tackle the problem, we produced transgenic pigs with the α-lactalbumin gene of cows inserted into their genome, which resulted in an increase in the lactose concentration in milk [109]. This significantly decreased the mortality rate among the piglets that were fed the modified milk. Another problem in swine breeding is the pollution of the environment with their feces, which contain high levels of phosphorus. This problem was resolved by producing transgenic pigs whose genome contained an inserted phytase-encoding gene of bacterial origin [110]. As a result, the level of phosphates in the feces of the transgenic pigs decreased by 75%.

Resistance to diseases is another extremely important aspect in the application of transgenesis in agriculture. Thus, the losses inflicted by mastitis (inflammation of the mammary gland caused by bacterial infection) in cattle exceed 1.7 billion dollars a year in the USA alone [111]. Mastitis is typically caused by staphylococci. Lysostaphin, a powerful peptidoglycan hydrolase secreted by *Staphylococcus simulans, *exhibits a bactericidal effect against staphylococci, causing mastitis. Transgenic cows [69] whose milk contains lysostaphin at a concentration of 0.014 mg/ml have been obtained. It was demonstrated that such cows are characterized by increased resistance to staphylococcal infections.

Bovine spongiform encephalopathy (BSE, also known as mad cow disease) is the most lethal disease affecting cattle in countries of the Northern Hemisphere. Removal of the prion protein gene that causes the disease was proposed as a way to combat it [112]. As a result, transgenic cows lacking the gene (and, thus, resistant to BSE) have been produced [113]. It is obvious that the use of such cows can reduce the incidence and spread of the disease epidemics.

These examples demonstrate that the use of TAs in agriculture is highly promising. The main restriction to the widespread distribution of TAs is the fear of the wider public regarding the safety of transgenic food products. More stringent regulatory requirements are imposed as a result, making it difficult to obtain permission to use TAs. In 2009 (the current edition from May 17, 2011), after more than 10 years of development, the FDA approved a procedure for considering applications for using TAs [114]. The procedure for the approval of new products is simpler in developing countries, and both the government and the public view TAs as one of the ways to resolve the problem of food security and improvement of living standards. As a result, most of the projects on the applications of TAs in agriculture are currently being implemented in countries, such as Brazil, Argentina, and China.

## CONCLUSIONS

Efficient methods for producing TAs expressing RPs have been developed over the past 20 years. TAs offer opportunities to significantly reduce costs in producing mAb and human RPs with post-translational modifications that closely match those of human proteins.

Until recently, the main reasons behind the reluctance to produce RPs using TAs in developing countries included a lack of developed laws regulating the use of TAs, strict ethical standards, and protests in the public against the use of animals as bioreactors.

However, the situation has begun to change. Detailed regulations to accompany the use of TAs for the production of RPs have been developed. The establishment of two manufacturing productions of RPs in the milk of TAs approved by regulatory agencies in the USA and EU has removed many issues related to the organization of production, while the expiration of patents on many biological preparations has increased competition between manufacturers, forcing companies to search for the most economically efficient technological models of production. Thus, it is very likely that in the near future the use of TAs in the biotechnology and food industries will expand.

## Abbreviations

mAb: monoclonal antibodies
MI: intranuclear microinjection of DNA
NT: nuclear transfer
RP: recombinant protein
RHA: recombinant human albumin
rhBChE: recombinant human butyrylcholinesterase
TA: transgenic animal
FDA: United States Food and Drug Administration
EMEA: European Medicines Evaluation Agency
CHO: cells – Chinese hamster ovary cells
ES cells: embryonic stem cells
UTR: untranslated region of a gene
